# Divorce and Suicide

**Published:** 1898-10

**Authors:** 


### Divorce and Suicide.

Since Prof. Lombroso advanced his theory declaring that genius is a form of degeneracy, an Italian school has sprung up, the apostles of which attempt to combine science and statistics. The latest theorist is Dr. Morselli, and he attempts to prove that suicide and divorce are closely related, and that, in fact, divorce is the chief cause of suicide. He finds that in Germany, where suicides are more frequent than in any other country, out of a total of 620 cases in a certain decade, 61 married women, 87 single women, 124 widows and 348 divorced women committed suicide. That is to say, more than one-half the suicides among women in Germany were among those divorced. Out of 4,000 male suicides, 204 were married men, 274 unmarried, 888 widowers, and 2,644 divorced. In both these investigations those married furnished the least number of suicides and those divorced the largest number. Further, in Ireland, where divorce is practically unknown, the ratio of suicides is less than in any other country, and in Denmark, where the proportion of divorces is highest, the proportion of suicides to the population is also highest.—*Journal of Medicine and Science.*

